# Moralized Health-Related Persuasion Undermines Social Cohesion

**DOI:** 10.3389/fpsyg.2018.00909

**Published:** 2018-06-12

**Authors:** Susanne Täuber

**Affiliations:** Department of Human Resource Management & Organizational Behavior, University of Groningen, Groningen, Netherlands

**Keywords:** persuasion, moralization, categorization, solidarity, social exclusion, unequal treatment

## Abstract

Integrating theory and research on persuasion, moralization, and intergroup relations, the present research aims to highlight the far-reaching impact of health-related persuasion on society. I propose that governments’ health-related persuasion leads to the emergence of new social norms, and in particular moral norms. Importantly, moral norms provide strong behavioral imperatives and are seen as binding for group members. This suggests that moralized persuasion has a strong potential to divide society along the lines of citizens who conform to and citizens who deviate from health-related moral norms. Thus, departing from the traditional focus on targets of persuasion, the present research focuses on those holding a moralized view on health and lifestyle. Key aspects of social cohesion as defined by the OECD (2011) have been tested across four studies. The main hypothesis tested is that those conforming to the norm (e.g., non-smokers, normal weight people, people with healthy lifestyles) will stigmatize those deviating from the norm (e.g., smokers, overweight people, people with unhealthy lifestyles). Flowing from stigmatization, less inclusion, lower solidarity with and greater endorsement of unequal treatment of those deviating from the moral norm are predicted. Four survey studies (total *N* = 1568) examining the proposed associations among non-smokers, normal weight people, and employees with healthy lifestyles are presented. The studies provide unanimous support for the hypothesis, with meta-analysis providing further support for the reliability of the findings. Consistent across studies, social cohesion indicators were negatively affected by health moralization through stigmatization of those deviating from health-related moral norms. Findings highlight an under-acknowledged potential of moralized health-related persuasion to divide society, thereby undermining cohesion and the achievement of important societal goals. In the discussion, limitations and relevant routes for future research are highlighted. Recommendations are derived for policy makers, institutions, employers, and individuals.

## Introduction

Persuasion is an important part of our lives. We are confronted with messages to make us buy a certain product, vote for political parties, or live our life in a certain way. At the same time, we try to persuade the people around us constantly, trying to convince them of our own opinion, to drop certain unhealthy habits, or to join certain groups. Given its pervasiveness, it is not surprising that the study of persuasion lies at the heart of diverse research areas, ranging from social psychology and the political sciences to advertising and marketing research (Knowles and Linn, 2004). Thus far, research and theorizing concerning persuasion overwhelmingly focused on those targeted by the persuasive message (e.g., Petty and Cacioppo, 1986; Olson and Zanna, 1993; Wood, 2000). The question how persuasion affects those already complying with the desired behavior or attitude has received little to no attention. I propose that this question is particularly relevant when persuasion is moralized and addresses large collectives of people, because in such contexts, persuasion likely leads to the emergence of new moral norms. In line with this proposition, the aim of this paper is threefold. The first aim is to situate persuasion in the realm of emergent moral norms, thereby carving out its relevance for intra- and intergroup dynamics. In order to achieve this aim, the present paper diverges from the traditional focus of considering the effects of persuasion on the targets, focusing instead on those already complying with the behavior at the heart of the persuasive message. The second aim is empirically test the key hypothesis derived from the above perspective, namely that those complying with a moralized health-related norm will be less inclusive toward, express less solidarity with, endorse unequal treatment of, and be more exclusionary and discriminatory toward those not complying with this norm. Finally, based on the theoretical rationale and empirical findings presented, the third aim is to derive practical recommendations as well as suggestions for relevant future research.

### Persuasion Can Lead to the Emergence of Moral Norms

When thinking about persuasion, the most prominent examples coming to mind probably concern marketing, where the acquisition of certain products stands central. Research shows that people are equipped with a range of strategies to resist such messages (e.g., Sagarin et al., 2002; Knowles and Linn, 2004; Fransen et al., 2015). Knowles and Linn (2004) contend that resistance is a precondition for persuasion, because “without resistance, persuasion, like preaching to the choir, is unnecessary babble” (p. vii). Importantly, when marketing is concerned, strategies to resist persuasion are commonly conceived of as properties of a person (e.g., Fransen et al., 2015). For instance, White and Dahl (2007) show that consumers have negative associations with brands that are used by people they do not wish to be associated with. Thus, the brand of the car one drives, the soft drink one prefers or the phone one uses can serve as the basis for distinguishing oneself from other individuals (Hollenbeck and Kaikati, 2012).

A more recent development is governments’ efforts to persuade entire populations to change their lifestyles. While being similar to marketing in its aim to achieve attitude and behavioral change, one distinct feature differentiating it from other forms of persuasion is its potential for creating new social norms. Specifically, rather than providing categorizations based on individual preferences such as concerning one’s car or phone, this form of persuasion leads to categorization based on conformity to or deviance from a societal norm. This also implies that resisting or being persuaded by such a message will no longer be perceived as a property of an individual, but as a property of a group member. Thus, while the car one drives or the phone one uses will be perceived as a matter of personal choice and preference, conformity to or deviance from an emerging group norm will be perceived as a matter of one’s relation with the group (Ellemers and van den Bos, 2012). As a consequence, effects of persuasion will be manifest not only at the individual, but also at the group level.

Moreover, persuasion concerning lifestyle and health will not evoke just any norm. For several reasons which I will detail below, persuasion concerning health and lifestyle is likely to evoke a *moral* norm. Consider the example of the Dutch government, which announced the transition of the traditional welfare state to a so called participatory society in the address of the King (Troonrede, 2013). In the note to the people, a strong focus is put on own responsibility for living and aging healthily. At the same time, harm caused to society by not living healthy is reiterated in public discourse and the media. This is done in particular by highlighting that money spent on health care costs cannot be spent on better education or infrastructure (Verschoor, 2015). Thus, not only are those living unhealthily depicted as harming society by incurring costs, these costs are also framed as a zero-sum conflict of interest. Prior research demonstrated that a focus on own responsibility and harming others are elements of communication that evoke considerations of the issue in questions in terms of morality (Rozin, 1999; Mulder, 2008). Further, salience of zero-sum conflicts of interest is an important element of moral exclusion (Opotow, 1990).

### The Specificity of Moral Norms

Before considering the impact of moral norms on relations between people in more detail, it is important to critically reflect on the general assumption that public debate and governmental persuasion can lead to the emergence of new moral norms. We are inherently social beings and our survival depends on living in groups (De Waal, 1996). Moral norms are a crucial tool to reinforce behavioral norms within groups (Ellemers and van den Bos, 2012). Such norms do not, by any means, exclusively concern universal morals. Rather, different groups can simply label certain behaviors as morally right or wrong, thereby regulating individual group members’ behavior in an effective way (Ellemers and van den Bos, 2012). Thus, a behavior that is considered moral by one group might be considered neutral or even immoral by another group (Ellemers and van den Bos, 2012). This surprisingly arbitrary nature of moral norms has been demonstrated repeatedly in a variety of studies using different approaches. For instance, Van Bavel et al. (2012) showed that people are capable of construing diverse issues in moral and non-moral terms, flexibly shifting their evaluation of the issue on a trial-to-trial basis. Similarly, Wright et al. (2008, Study 1) asked participants to classify 40 diverse issues ranging from exercising over honesty to the death penalty as moral or non-moral. None of those issues was unanimously classified as moral, and only one was classified as non-moral by all participants.

The review above suggests that virtually any issue or behavior can be perceived as a moral imperative, simply based on individual convictions, group norms, or experimenter instructions. But issues can also lose or gain moral connotations over the course of time, a process called moralization when an issue is increasingly viewed as moral or amoralization when an issue is increasingly viewed as non-moral. Rozin (1999) described these processes using cigarette smoking (Rozin and Singh, 1999) and vegetarianism (Rozin et al., 1997) as examples for issues that shifted from being perceived as an individual choice to being moralized in the past decades. Relatedly, divorce and homosexuality shifted from being moralized to being perceived as a personal preference over the past decades (Rozin, 1999). Interestingly, Rozin (1999, p. 220) observed that people often try to establish claims of new moral values, but that “most such claims fade away without producing much of a ripple.” Thus, why would health and lifestyle emerge as a new moralized norm rather than fading away? The answer is probably that health and lifestyle involve all the factors that have been identified as facilitating moralization. Factors facilitating successful moralization are perceptions that the behavior in question is under people’s self-control and they are thus responsible for showing or not showing the behavior and that the behavior elicits unwarranted harm on others (Rozin, 1999; Mulder, 2008). In addition to this, Rozin (1999) contends that lasting moralization is particularly likely for behaviors associated with stigmatized and marginalized groups. In light of those facilitating factors, health appears to be a prime candidate for moralization. With healthy and fit bodies being seen as indicative of self-control, self-denial, and willpower (Bisogni et al., 2002), self-control is an issue deeply ingrained in the health domain. Public debate focusing on citizens’ responsibility to live healthily underlines the (presumed) controllability of health outcomes. Moreover, unhealthy lifestyles and poor health outcomes are disproportionately associated with and statistically more prevalent among those with lower socio-economic status and those with a migratory background (e.g., Adler and Ostrove, 1999). Put differently, unhealthy lifestyles and poor health outcomes demonstrate a link with stigmatized and marginalized groups, thereby fulfilling another factor that makes moralization of health more likely. Finally, framing poor health outcomes in terms of incurring costs on society makes harm inflicted on the collective salient. All these factors feed into health being an exceptionally well-suited domain for lasting moralization on a societal level. Importantly, once a behavior has become moralized, it is perceived as binding for the group (Ellemers and van den Bos, 2012). Thus, whether one is deemed a respected group member with an esteemed social image depends on whether one conforms to the moral norms of the group one belongs to. The next section considers the impact of moral norms on social relationships in more detail.

### The Impact of Moral Norms on Intra- and Intergroup Processes

Moral norms are imperative for our living in groups (De Waal, 1996) and group identity is defined by shared moral norms (Ellemers and van den Bos, 2012). Moral norms dictate what “good” people should do (Haidt, 2001), and consequently regulate individual group members’ behavior in groups, their standing within the group, and how respected they are (Ellemers et al., 2008). It follows logically that those violating a moral norm seen as binding and defining for the group cannot, by definition, be good group members. This division into moral and immoral others might be considered functional from an evolutionary point of view. For instance, others’ morality is considered particularly relevant for survival (Wojciszke, 1994, 2005; De Waal, 1996) and humans indeed appear to be particularly sensitive to cues of immorality in others (Gantman and Van Bavel, 2014, 2015). This aligns with Error Management Theory (Haselton and Buss, 2000), which posits that in the moral domain, false positives (incorrectly assuming the other is moral) are costlier than false negatives (incorrectly assuming the other is immoral). Further, negative behaviors are perceived as more diagnostic than positive behaviors in the moral domain, while this pattern is reversed in the competence domain (Skowronski and Carlston, 1987, 1989). Thus, not only will people be more alert concerning indicators of immorality in others, once they have actually found such indicators, these will be incredibly difficult to correct for (Skowronski and Carlston, 1992). Finally, drawing on the functional perspective, recent research demonstrated that morality is another fundamental social category along which people spontaneously categorize others (van Leeuwen et al., 2012).

The above considerations suggest that generally, dividing one’s social environment into those conforming to and those deviating from moral norms is functional and benefits our survival and successful group living. However, in conjunction with the finding that virtually any behavior can be construed in moral terms, such a division can easily emerge from more or less arbitrary features. In such cases, I would argue that the negative consequences of dividing the world into morally good and morally bad people outweigh the potential benefits thereof. This is because being considered immoral has much more serious consequences than being considered, for instance, incompetent. These insights are based on different lines of theory and research as introduced above, but also on extensive research into moral convictions conducted by Skitka et al. (2005) and Skitka (2010). Moral convictions refer to “a strong and absolute belief that something is right or wrong, moral or immoral” (Skitka and Mullen, 2002, p. 36). While moral convictions have positive potential in that they are major motivational catalysts for social action (van Zomeren et al., 2011, 2012; Täuber et al., 2015), there is also a dark side to them, as discovered over the past years. For instance, people have been shown to perceive their moral convictions as imperative for others’ behavior and are unwilling to compromise (Skitka et al., 2015). Naturally, this has consequences for how people view others who hold divergent attitudes. Because moral issues are seen as a non-negotiable truth, they generate an expectation of consensus from others – thus, that everybody should agree with me (Wright et al., 2013). It is this expectation which, according to Wright et al. (2013, p. 37), “makes anyone who disagrees an outsider: an outgroup member worthy of rebuke.” Consistent with this, Haidt et al. (2003) showed that college students, while generally valuing demographic diversity, reported significantly lower desire to interact with other who differed from them regarding moral values. These findings suggest that, to the extent that people view a behavior in moral terms, they are likely to stigmatize those not complying with the behavior. More specifically, viewing health as a moral issue is expected to be associated with greater expression of negative attitudes toward those who are seen as transgressing the moral norm. Stigmatization processes refer to the ascription of negative attributes to individuals, which are often behaviourally followed by social distance, discrimination, and exclusion (e.g., Link and Phelan, 2001; Sikorski et al., 2015).

Similar to research on stigmatization (e.g., Link and Phelan, 2001; Sikorski et al., 2012, 2015), also research into moral convictions demonstrates a strong association between attitudes and behavior. For instance, moral mandates – which refer to the readiness to take action based on moral convictions – have been shown to be used to justify “any number of extreme actions” in order to achieve the desired outcome (Skitka and Mullen, 2002, p. 39). These findings do not hold for moral convictions alone. Wright et al. (2008) showed that simply believing an issue to have a moral connotation made people more intolerant toward others with a diverging attitude. Relatedly, Van Bavel et al. (2012) concluded that, compared to non-moral evaluations, moral evaluations of the same action were faster, more extreme, and more strongly associated with the belief that absolutely nobody or everybody should engage in this action. In sum, research consistently demonstrates that attitudinal divergence on moral, as compared to non-moral, issues elicits higher intolerance (Haidt et al., 2003; Skitka et al., 2005; Wright et al., 2008). Such intolerance involves greater preferred social and physical distance from attitudinally dissimilar others (Haidt et al., 2003; Wright et al., 2008), more extreme evaluations (Van Bavel et al., 2012), lower levels of good will and cooperativeness in attitudinally heterogeneous groups, and a greater inability to generate procedural solutions to resolve disagreements (Skitka et al., 2005).

Summarizing the above, the moralization of health might thus undermine important indicators of social cohesion. According to the OECD (2011), social cohesion is highly relevant for inclusive growth and societies are considered cohesive when they are “… stable, safe and just, and are based on the promotion and protection of all human rights, as well as on non-discrimination, tolerance, respect for diversity, equality of opportunity, solidarity, security and participation of all people, including disadvantaged and vulnerable groups and persons.” (OECD, 2011, p. 53). The measures across the three studies reflect different aspects of this definition by focusing on *participation of all people, including disadvantaged and vulnerable groups and persons* (Study 1), on *solidarity and equality of opportunity* (Studies 2a and 2b), and on *non-discrimination, tolerance, and respect for diversity* (Study 3).

### The Present Research

The present research aims to complement and extend prior theorizing and research in several ways: First, going beyond obesity-specific stigmatization and discrimination (Puhl and Brownell, 2001; Puhl and Luedicke, 2012; Flint and Snook, 2014; Sikorski et al., 2015; Flint et al., 2016), the current research proposes and tests that diverse health-related behaviors, to the extent that they have a moral connotation, can and will serve to divide people. Second, going beyond consequences of moral convictions for interindividual interactions (e.g., Haidt et al., 2003; Wright et al., 2008; Skitka et al., 2015), the current research proposes and tests important intergroup dynamics which are thought to result from moralization, and which are thought to undermine social cohesion. In the following, I present results from four survey studies that measured health moralization, stigmatization, and different aspects of social cohesion. All studies tested the same basic hypothesis, namely that health moralization undermines social cohesion through stigmatization of others who do not conform to a health-related norm. Study 1 was conducted among a student sample, Studies 2a and 2b among adult samples, and Study 3 among employees. Below, I provide a detailed overview over the measures, after which I will elaborate on each study’s method in terms of recruitment, response rate, and compilation of the sample reported here. Finally, I will present descriptive statistics, and the results of the mediation analyses testing the key hypothesis for all studies. I will conclude by summarizing the results using meta-analysis following Goh et al. (2016).

### Overview Over the Variables

#### Health Moralization

The extent to which respondents perceived health as a moral issue was examined in all studies. The wording of the items varied slightly across studies in order to adapt the items to the respective study contexts. However, the essence of the items was the same in all studies, namely whether respondents thought of health as something that is obligatory and as harming others when not conformed to (Rozin, 1999; Mulder, 2008). Appendix A provides an overview over the variables used in the studies reported below.

#### Stigmatization

The extent to which respondents stigmatized, thus ascribed negative traits to, others who deviate from the health norm, was examined in all studies. The wording of the items varied across studies according to the respective study contexts. These items were adapted from prior research on stigmatization, specifically from Crandall’s (1994) work on fat stigma. Stigmatization referred to smokers in Studies 1 and 2a, to people with overweight in Study 2b, and to people with unhealthy lifestyles in Study 3.

#### Social Cohesion

Building on the OECD’s (2011) definition of social cohesion, different aspects of social cohesion were measured across studies. Study 1 focused on inclusion, Studies 2a and 2b focused on solidarity and equal treatment, and Study 3 focused on inclusion, non-discrimination, and non-exclusion. Below I introduce the measures in detail.

In Study 1, the focus was on inclusion. *Social inclusion* is often measured in terms of preferences for individual interaction, for instance by asking respondents whether they would agree having an obese person marrying into their family, or having a mentally ill person living in their neighborhood (Sikorski et al., 2015). Given the aim of the current research to illustrate the impact of health moralization on a societal level, however, Study 1 operationalized social inclusion in terms of respondents’ perceived overlap between those conforming to and those deviating from the moral norm, as well as between society and both groups of citizens using the intergroup item of the Inclusion of Others into the Self scale (IOS, Schubert and Otten, 2002). After extensive testing, this item was found to be an “easily applicable, comprehensive, and very sensitive assessment of the salient self-categorization at a social level” (Schubert and Otten, 2002, p. 373). The item metaphorically maps intergroup relations onto the spatial dimension, thereby capturing central aspects of relations between groups in society. Specifically, the pictorial IOS was used to measure perceived overlap between smokers and non-smokers, smokers and society, non-smokers and society, ill people and healthy people, ill people and society, as well as healthy people and society (from 1 = no overlap at all, to 7 = almost complete overlap). Exploratory factor analysis (EFA) with principal axis factoring (PAF) with direct oblimin rotation revealed four factors with eigenvalues > 1. The total variance explained was 47.85%. The moralization items loaded on one factor (9.07% explained variance), and the anti-smoker attitudes items (22.45% explained variance) loaded on another factor. The IOS items (reflecting the dependent variable) loaded on two factors. Specifically, the items referring to inclusion of smokers/ill people with non-smokers/healthy people/society loaded on one factor (12.48% explained variance), while the items referring to inclusion of non-smokers/healthy people and society loaded on another factor (3.85% explained variance). Based on these results, the following scales were computed: Moralization of health (α = 0.73), anti-smoker attitudes (α = 0.79), inclusion of deviants (α = 0.74) and inclusion of conformers (*r* = 0.52, *p* < 0.001).

In Studies 2a and 2b, the focus was on solidarity and equal treatment. Three constructs were measured, namely respondents’ *willingness to show solidarity*, their *expectation of others showing solidarity*, and their endorsement of unequal treatment of deviants from the health norm. Specifically, respondents were asked to indicate to what extent they thought different groups of deviants should *pay more health insurance* compared to conformers to the health norm. On a seven-point scale, 1 indicated ill people, smokers, overweight people, and people with unhealthy lifestyles, 4 reflected respondents’ perception that both groups should pay an equal amount, and 7 indicated that healthy people, non-smokers, people with normal weight, and people with healthy lifestyles should pay more. This measure was mean-centered, such that the resulting scale reflected the endorsement of equal payment by zero, and endorsement of deviants paying more by higher values. Overall, the measured constructs were thus coded in a way that higher values reflect greater (expectations of) solidarity and greater endorsement of equality.

For both studies, EFA with PAF with direct oblimin rotation revealed five factors with eigenvalues > 1. Two items loaded on the factor health moralization (2.59 and 6.32% explained variance for Study 2a and 2b, respectively), three items on anti-smoker and anti-fat attitudes (6.18 and 10.61% explained variance for Study 2a and 2b, respectively), four items on willingness to show solidarity (8.83 and 27.16% explained variance for Study 2a and 2b, respectively), three items on expectations of solidarity (14.09 and 13.16% explained variance for Study 2a and 2b, respectively), and four items on the factor pay more (28.29 and 3.01% explained variance for Study 2a and 2b, respectively). Based on these results, scales were computed for health moralization (*r* = 0.40, *p* < 0.001 and *r* = 0.40, *p* < 0.001 for Study 2a and 2b, respectively), anti-smoker attitudes (α = 0.74, Study 2a), anti-fat attitudes (α = 0.79, Study 2b), willingness to show solidarity (α = 0.87 and α = 0.86 for Study 2a and 2b, respectively), expectation of others showing solidarity with self (α = 0.86 and α = 0.79 for Study 2a and 2b, respectively), and respondents’ endorsement of unequal treatment of deviants from the health norm (α = 0.79 and α = 0.79, for Study 2a and 2b, respectively).

In Study 3, the focus was on inclusion and equal treatment. Respondents’ perception that colleagues with healthy and unhealthy lifestyles belong to the same rather than to different groups was measured with four items adapted from Gaertner et al. (1993). In order to avoid social desirability effects, respondents were not asked whether they engage in discrimination and exclusion themselves. Instead, respondents were asked to indicate their agreement with statements such as “Colleagues with unhealthy lifestyles are often treated unfairly in the work place.” Perceived discrimination and exclusion of colleagues with unhealthy lifestyles were measured with five and three items (adapted from Kesseler et al., 1999 and Morrison et al., 1999, respectively). In order to keep interpretation of the constructs comparable across studies, the items referring to discrimination and exclusion were reverse coded. Thus, higher values of the scales in this study reflect the perception of being one group (categorization), of equal treatment (discrimination recoded), and of inclusion (exclusion recoded). EFA with PAF with direct oblimin rotation revealed five factors with eigenvalues > 1. Four items loaded on the factor health moralization (8.06% explained variance), seven items on negative attitudes toward people with unhealthy lifestyles (34.77% explained variance), five items on discrimination (4.65 explained variance), four items on categorization (5.89% explained variance), and three items on exclusion (3.80% explained variance). Based on these results, scales were computed for health moralization (α = 0.75), negative attitudes toward colleagues with an unhealthy lifestyle (α = 0.85), perceived discrimination of colleagues with unhealthy lifestyles (α = 0.86), categorization (α = 0.78), and perceived exclusion of colleagues with unhealthy lifestyles (α = 0.86).

## Materials and Methods

### Study 1

The study was conducted in the lab of the author’s faculty. In order to not discriminate, the study was not advertised as inviting non-smokers in particular. In total, 271 students came to the lab for participation in the study in exchange for course credit. Respondents were seated in separate cubicles where they were presented with the questionnaire through a computer. Respondents were informed that the survey was concerned with their opinions about health and lifestyle, that participation in the study was voluntary, that their individual responses would be completely anonymous and that filling in the questionnaire would take approximately 10 min. Based on this information, respondents were asked to give informed consent. Before proceeding to the questionnaire, respondents answered the following question: “Do you consider yourself a smoker or a non-smoker?” (1 = “I consider myself a smoker,” 2 = “I consider myself a non-smoker”). Based on respondents’ self-reported categorization, only those who considered themselves non-smokers were presented with the measures reported here. Respondents who identified as smokers (*N* = 39) were redirected to a study that investigated the extent to which those who deviate from a health norm (i.e., smokers) feel morally judged by others and how this affects their motivation to conform to the health norm (i.e., quit smoking) in the future. Two-hundred-thirty-two students self-identified as non-smokers (135 female, 97 male; *M_age_* = 22.22, *SD_age_* = 2.70) and filled in the questionnaire as presented above.

### Study 2a

Respondents were recruited from a panel about public transport. A randomly selected sample of 4310 people from this panel received an invitation to participate in a survey about health and lifestyle. The invitation explicitly stressed that this survey was conducted by the author’s university and that participation was voluntary. The questionnaire could be accessed through a link provided in the email. The study was presented using the online survey tool Qualtrics. Upon starting the study, respondents were informed that their individual responses would be completely anonymous and that filling in the questionnaire would take approximately 10 min. Based on this information, respondents were asked to give informed consent before proceeding to the questionnaire. In total, 1100 people started the questionnaire, reflecting a response rate of 25.52%. Before proceeding to the measures, respondents answered the following question: “Do you consider yourself a smoker or a non-smoker?” (1 = “I consider myself a smoker,” 2 = “I consider myself a non-smoker”). Based on respondents’ self-reported categorization, only those who considered themselves non-smokers were presented with the measures reported here. Respondents who identified as smokers (*N* = 112) were redirected to a study that investigated the extent to which those who deviate from a health norm feel morally judged by others and how this affects their motivation to conform to the health norm in the future. The results for this sample are currently being written up. Of the 955 respondents who identified as non-smokers, 189 filled in less than one third of the questionnaire and were therefore disregarded for data analysis. This resulted in a sample of 766 non-smokers (365 female, 401 male; *M_age_* = 57.85, *SD_age_* = 14.17), whose answers are reported here.

### Study 2b

Respondents were recruited from a panel about public transport. A randomly selected sample of 4310 people from this panel received an invitation to participate in a survey about health and lifestyle. The invitation explicitly stressed that this survey was conducted by the author’s university and that participation was voluntary. The questionnaire could be accessed through a link provided in the email. The study was presented using the online survey tool Qualtrics. Upon starting the study, respondents were informed that their individual responses would be completely anonymous and that filling in the questionnaire would take approximately 10 min. Based on this information, respondents were asked to give informed consent before proceeding to the questionnaire. In total, 1300 people started the questionnaire, reflecting a response rate of 30.16%. Before proceeding to the measures, respondents answered the following question: Would you consider yourself as …” (1 = A person with normal weight, 2 = A person with a little overweight, 3 = A person with overweight, 4 = A person with a lot of overweight). Of the sample, 455 (43.1%) identified as persons with normal weight, 352 (33.3%) identified as persons with a little overweight, 212 (20.1%) identified as persons with overweight, and 37 (3.5%) identified as person with a lot of overweight. Respondents who identified as persons with overweight and a lot of overweight (*N* = 249) were redirected to a study that investigated the extent to which those who deviate from a health norm feel morally judged by others and how this affects their motivation to conform to the health norm in the future. The results for this sample are currently being written up. For the purpose of the present paper, a data-file was compiled of respondents self-identifying as having normal weight and a little overweight, and from this sample, only those respondents were included in the data analysis who were considered as having normal weight based on a BMI smaller than 25. This cut-off value left *n* = 447 respondents for data analysis. Note that of these 447 respondents with objectively normal weight based on BMI < 25, 74 (16.6%) categorized themselves as having a little overweight. Similarly, of the 265 respondents who were objectively overweight as indicated by a BMI > 25, 35 (8.6%), categorized themselves as a person with normal weight. The final sample consisted of 447 respondents with normal weight (BMI < 25; *N* = 447; 223 female, 224 male; *M_age_* = 56.43, *SD_age_* = 14.94).

### Study 3

The study was conducted among German employees. Respondents were recruited through a snowball system using social media and the professional network of a research assistant. In total, 293 people started the questionnaire. Respondents were informed that the survey was concerned with their opinions about health and lifestyle at work, that participation in the study was voluntary, that their individual responses would be completely anonymous and that filling in the questionnaire would take approximately 10 min. Based on this information, respondents were asked to give informed consent before proceeding to the questions. Respondents filling in less than one third of the questionnaire were disregarded for analysis. Note that in Study 3, respondents’ status in terms of conforming to or deviating from the norm of having a healthy lifestyle was more ambiguous than in Studies 1, 2a, and 2b because no pre-screening was employed. In order to minimize this ambiguity and maximize comparability of Study 3 with the previous studies, additional analyses were carried out. Specifically, respondents indicated whether they considered their own lifestyle as healthy on a five-point scale from 1 (very unhealthy) over 3 (neither unhealthy nor healthy) to 5 (very healthy). On average, respondents considered their lifestyle as healthy (*M* = 3.63, *SD* = 0.74), which was significantly above the midpoint of the scale, *t*(189) = 11.84, *p* < 0.001. Of the overall sample, 123 respondents considered their lifestyle as healthy and very healthy, and 67 respondents considered their lifestyle as neither healthy nor unhealthy and as unhealthy. No respondent considered their lifestyle as very unhealthy. In order to keep the comparability between studies intact, only respondents considering their lifestyles healthy and very healthy were retained for analysis, resulting in a final sample of 123 respondents (82 female, 41 male; *M_age_* = 36.80, *SD_age_* = 15.10; *M_tenure_* = 8.42, *SD_tenure_* = 10.92).

## Results

### Descriptive Statistics

**FIGURE 1:**
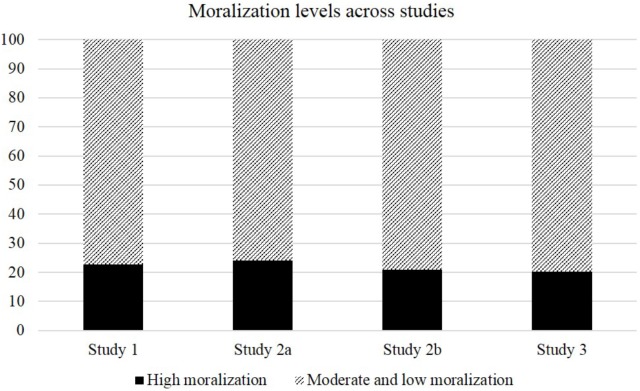
Prevalence of strong vs. moderate to low health moralization across studies.

Descriptive statistics for moralization, negative attitudes toward those who deviate from a health norm and indicators of social cohesion can be found in **Table 1**. In general, levels of moralization were similar across studies. In order to gain more insights into the prevalence of moralization, I have treated agreement and strong agreement with the statements referring to health moralization as high levels of moralization; neutral, disagreement and strong disagreement were treated as moderate to low levels of moralization. This results in very similar percentages of respondents strongly moralizing health across studies, namely 22.80, 24.00, 21.00, and 20.30% in Studies 1, 2a, 2b, and 3, respectively. **Figure 1** provides an overview over the proportions.

**Table 1:** Descriptive statistics for Moralization, Stigmatization, and Social Cohesion indicators.

	Study 1	Study 2a	Study 2b	Study 3
	
	*M (SD)*	*M (SD)*	*M (SD)*	*M (SD)*
*Moralization*	3.16 (0.78)	3.13 (0.81)	3.08 (0.83)	3.20 (0.81)
*Stigmatization*	2.90 (0.70)	2.85 (0.80)	2.55 (0.73)	2.27 (0.79)
**Social Cohesion Indicators**			
*InclusionD^a^*	4.74 (1.12)			
*InclusionC^b^*	5.84 (0.97)			
*Solidarity*		2.51 (0.79)	2.43 (0.80)	
*Expectation*		3.66 (0.72)	3.71 (0.64)	
*Equal Payment^c^*		1.73 (0.86)	1.75 (0.87)	
*Categorization*				3.94 (0.71)
*Equal Treatment*				4.01 (0.78)
*InclusionD^a^*				3.97 (0.77)

*^a^Inclusion of those deviating from the health norm; ^b^Inclusion of those conforming to the health norm; ^c^Mean indicates deviation from zero, with zero reflecting endorsement of equal treatment. Higher values indicate greater endorsement of deviants paying more than conformers.*

Levels of stigmatization as well as the social cohesion indicators were also similar across studies. In Study 1, respondents perceived those deviating from the health norm to be significantly less included with society and with their group of conformers than those conforming to the health norm, *t*(231) = -0.14.42, *p* < 0.001. In Studies 2a and 2b, respondents’ level of wanting to show solidarity was somewhat lower than their expectations of solidarity. This makes sense theoretically, since respondents are conforming to the health norm, and thus might feel entitled to solidarity from others while feeling less compelled to showing solidarity with those deviating from the health norm. However, the different mean levels might also simply be due to the fact that the targets of the questions differed; while showing solidarity referred to solidarity with those deviating from the health norm in particular, expecting solidarity referred to “others” in general. Respondents in both Study 2a and 2b endorsed higher payments of deviants significantly more than equal payment, *t*(765) = 55.93, *p* < 0.001 and *t*(446) = 42.17, *p* < 0.001. Indicators of social cohesion in Study 3 showed similar levels as those in the other studies.

### Mediation Analyses

In order to test the key hypothesis of this research that health moralization impairs social cohesion through stigmatization of those deviating from the health-related norm, simple mediations were run with z-standardized variables using model 4 with 5000 re-samples (pre-defined, PROCESS macro, Hayes, 2012). For ease of exposition and in order to avoid alpha error accumulation, the social cohesion indicators were collapsed into one scale in those studies that used more than one indicator. This was done in such a way that higher values in social cohesion indicators reflect more inclusion, more solidarity, and more equal treatment. Specifically, Study 1 had only inclusion as indicator and thus remained unchanged. Studies 2a and 2b had three indicators, of which two referred to solidarity and one referred to equal treatment. Collapsing across those three indicators resulted in a reliable scale of 11 items (α = 0.81 and α = 0.81 in Study 2a and 2b, respectively). Higher values of the resulting scale reflect greater social cohesion. Study 3 had three indicators of social cohesion, of which two referred to exclusion and one referred to unequal treatment. Collapsing across these three indicators resulted in a reliable scale of 12 items (α = 0.86). Higher values of the resulting scale reflect more inclusion and more equal treatment. The first relationship of interest was the mediator model, thus the relationship between health moralization and stigmatization (MS). **Table 2** provides an overview over the mediator model across studies. As can be seen, health moralization was associated with significantly greater stigmatization of those deviating from a health-related norm in all studies, irrespective of whether the norm referred to smoking, weight or lifestyle.

**Table 2:** Statistics for mediator model (relationship between moralization and stigmatization).

	*b^a^* (SE)	*t*	*F*(df)	*R^2^*
Study 1	0.21 (0.06)	3.22^∗∗^	10.40 (1,230)^∗∗^	0.043
Study 2a	0.45 (0.03)	13.99^∗∗∗^	176.94 (1,764)^∗∗∗^	0.188
Study 2b	0.36 (0.04)	8.76^∗∗∗^	76.68 (1,445)^∗∗∗^	0.147
Study 3	0.31 (0.09)	3.28^∗∗^	10.73 (1,120)^∗∗^	0.082

*^a^Z-standardized regression coefficients; ^∗^p < 0.05, ^∗∗^p < 0.01, ^∗∗∗^p < 0.001.*

The next relationship of interest was the dependent variable model, thus the association between moralization and social cohesion (MSC) when taking into account stigmatization as a potential mediator. **Table 3** provides an overview over the dependent variable model across studies. Specifically, social cohesion was significantly and negatively associated with stigmatization in all studies, irrespective of stigmatization referring to smokers, overweight people, or people with an unhealthy lifestyle. Health moralization remained a significant predictor of social cohesion in Studies 2a and 2b, but not in Studies 1 and 3. In accordance with these findings, a direct negative effect of moralization on social cohesion was evident in Studies 2a and 2b, *CI_95%_* (-0.294, -0.148) and *CI_95%_* (-0.329, -0.149), respectively. By contrast, no direct effect of moralization on social cohesion was evident in Studies 1 and 3, *CI_95%_* (-0.197, 0.055) and *CI_95%_* (-0.203, 0.078), respectively. Importantly, the hypothesized indirect effect of health moralization on social cohesion through stigmatization was evident in all studies. **Table 4** provides an overview over the corresponding statistics.

**Table 3:** Statistics for dependent variable model (relationship between moralization and social cohesion while controlling for stigmatization).

	*b^a^* (SE)	*t*	*F*(df)	*R^2^*
**Study 1**			
Moralization	-0.07 (0.06)	-1.11	13.00 (2,229)^∗∗∗^	0.102
Stigmatization	-0.30 (0.06)	-4.64^∗∗∗^		
**Study 2a**			
Moralization	-0.22 (0.04)	-5.97^∗∗∗^	76.82 (2,763)^∗∗∗^	0.168
Stigmatization	-0.26 (0.04)	-6.99^∗∗∗^		
**Study 2b**			
Moralization	-0.24 (0.05)	-5.22^∗∗∗^	33.09 (2,444)^∗∗∗^	0.130
Stigmatization	-0.18 (0.05)	-3.76^∗∗∗^		
**Study 3**			
Moralization	-0.18 (0.08)	-2.13^∗^	15.98 (2,119)^∗∗∗^	0.212
Stigmatization	-0.35 (0.08)	-4.40^∗∗∗^		

*^a^Z-standardized regression coefficients; ^∗^p < 0.05, ^∗∗^p < 0.01, ^∗∗∗^p < 0.001. Social cohesion indicators (dependent variables) are inclusion (Studies 1 and 3), solidarity (Studies 2a and 2b), and endorsement of equal treatment (Studies 2a, 2b, and 3).*

**Table 4:** Statistics for the indirect effect of moralization on social cohesion through stigmatization.

	*b^a^* (*SE*)	*CI_95%_ [LLCI, ULCI]*	*Z*
Study 1	-0.06 (0.02)	[-0.121, -0.027]	-2.61^∗∗^
Study 2a	-0.12 (0.03)	[-0.166, -0.064]	-6.24^∗∗∗^
Study 2b	-0.07 (0.02)	[-0.115, -0.028]	-3.43^∗∗∗^
Study 3	-0.11 (0.04)	[-0.217, -0.039]	-2.59^∗∗^

*^a^Z-standardized regression coefficients; ^∗^p < 0.05, ^∗∗^p < 0.01, ^∗∗∗^p < 0.001.*

### Meta-Analysis

**FIGURE 2:**
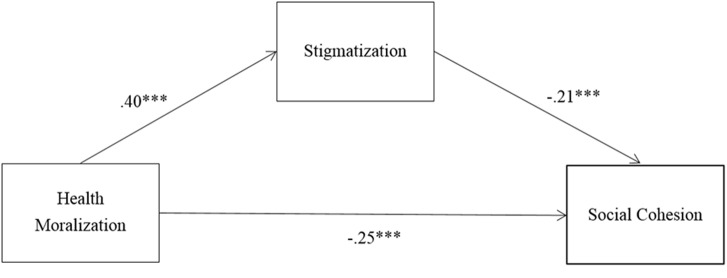
Summary of meta-analytic associations between the predictor, the mediator, and the dependent variable.

Finally, in order to provide a more comprehensive overview and to assess the overall reliability of the reported relationships, I conducted a mini meta-analysis following the recommendations of Goh et al. (2016). Specifically, separate analyses were performed for the relationship between the predictor and the mediator (health moralization and stigmatization), between the predictor and the dependent variable (health moralization and social cohesion) and between the mediator and the dependent variable (stigmatization and social cohesion, SSC). **Table 5** summarizes the findings. Fixed effects were used in which the mean effect size (in this case the mean correlation) was weighted by sample size. All correlations were Fisher’s *z* transformed (see *Mrz* in **Table 5**), and then converted back to correlations for presentation (see *Mr* in **Table 5**). Across the four studies, the associations between health MS, health MSC, and SSC were highly significant. This allows for some confidence in the findings reported here. **Figure 2** summarizes the meta-analytic associations between the predictor, the mediator, and the dependent variable.

**Table 5:** Associations of health moralization with stigmatization (MS) and social cohesion (MSC), and stigmatization with social cohesion (SSC).

	*N*	*MS*	*MSC*	*SSC*
Study 1	232	0.21^∗∗^	-0.07^∗^	-0.30^∗∗∗^
Study 2a	766	0.45^∗∗∗^	-0.22^∗∗∗^	-0.26^∗∗∗^
Study 2b	447	0.36^∗∗∗^	-0.24^∗∗∗^	-0.18^∗∗∗^
Study 3	123	0.31^∗∗^	-0.18^∗^	-0.39^∗∗∗^
*Mrz*		0.40	-0.21	-0.25
*Mr*		0.38	-0.21	-0.25
*Combined Z*		15.83^∗∗∗^	-8.25^∗∗∗^	-10.10^∗∗∗^
CI_95%_		0.338, 0.432	-.253, -0.158	-0.297, -0.204

*^∗^p < 0.05, ^∗∗^p < 0.001. Indicators of social cohesion were social inclusion (Study 1), willingness to be solidary, expectations of others showing solidarity, perception that deviants should pay more (Studies 2a and 2b), categorization, exclusion, and discrimination (Study 3). Mr = fixed-effects weighted mean correlation across studies. Mrz = Fisher’s-z transformed correlations for normalization. Combined Z = Summary p-value for all studies based on Stouffer’s Z test, together with confidence intervals.*

## Discussion

I started out with the proposition that persuasion concerning health and lifestyle is relevant for intra- and intergroup dynamics because it leads to the emergence of new moral norms on a societal level. Specifically, I suggested that public discourse about health that highlights own responsibility and harm inflicted on others, together with the strong association of health with self-control and the greater prevalence of poor health outcomes among marginalized groups in society all operate to facilitate lasting moralization of health (Rozin, 1999). Based on insights from moral psychology, I expected health moralization to be associated with stigmatization of citizens deviating from the moral health norm, and with decreased levels of social cohesion. Four studies provided converging support for this notion. The more those conforming to a health norm – non-smokers, normal weight people, and employees with healthy lifestyles – moralized health or lifestyle, the more they stigmatized those deviating from the norm – smokers, overweight people, ill people, and employees with an unhealthy lifestyle – which was associated with less inclusion, lower solidarity, and endorsement of unequal treatment. All of these outcomes are indicators of social cohesion, which is considered highly relevant for societies’ inclusive growth (OECD, 2011). The predicted processes were observed across samples varying in age, nationality, and context.

Findings align with a body of research demonstrating that obese people are stigmatized and discriminated against in numerous settings such as schools, public spaces, and the workplace (Puhl and Brownell, 2001; Puhl and Luedicke, 2012; Flint and Snook, 2014; Sikorski et al., 2015; Flint et al., 2016). However, they valuably extend prior theorizing and research by demonstrating processes undermining social cohesion on a variety of health-related aspects. Thus, going beyond obesity-specific stigmatization and discrimination, the main message of the current research is that any health-related condition or behavior that acquires a moral connotation can and will serve to divide people. Further, going beyond consequences of moral convictions for interindividual interactions (e.g., Haidt et al., 2003; Wright et al., 2008; Skitka et al., 2015), the current research highlights the intergroup dynamics flowing from moralization by stimulating a supposedly legitimate division of society into those conforming to and those deviating from emergent moral norms.

### Some Thoughts on Causality

While the findings reported here align with the theoretical rationale introduced, the employed research designs do not allow for causal inferences about the relationships under scrutiny. Based on prior theorizing about the consequences of moralization (e.g., Rozin, 1999), I have predicted that those who deviate from a moral norm will be perceived negatively, as reflected in increased stigmatization levels (e.g., Crandall, 1994). Increased levels of stigmatization, in turn, serve as a legitimization for unequal treatment and exclusion (e.g., Haidt et al., 1993; Wright et al., 2008; Sikorski et al., 2015). As outlined in the supplementary analyses, the mediation models work equally well when the mediator stigmatization and the dependent variable social cohesion are switched. What does this mean? Indeed, both processes are plausible. The reasoning guiding my research reflects decision-stage models of attribution (e.g., Heider, 1958), which conceive of observers as “naïve scientists.” In an attempt to arrive at conclusions about the causality of events, observers are thought to first assign controllability, which is strongly associated with morality – if an outcome is considered controllable, someone failing to achieve the outcome will be seen as immoral (e.g., Weiner, 1986). In a second step, observers assign responsibility, which is reflected in stigmatization. In the final stage, blame is assigned, which is reflected in exclusion, little solidarity, and discrimination as described in the present research.

However, stigma researchers have also suggested that – in particular in non-laboratory contexts – people might not necessarily go through the stages in order (Shaver, 1985). This is because “an attribution of moral responsibility is a social judgment based on personal ethical standards against which the agent’s behavior is measured” (cf. Mantler et al., 2003, p. 142). Put differently, when an observer holds specific ethical or moral standards, they might hold strong preexisting bias (Alicke, 2000; cf. Mantler et al., 2003). They would then first focus on the blameworthiness of the agent’s deviating behavior. In this case, observers would first “decide” that, for instance, a smoker or an overweight person should pay more health insurance. Only then would observers go backward through the stages, so as to confirm their judgment and their preexisting personal bias, for instance by thinking that smokers and overweight people are lazy and lacking self-control. Thus, stigmatization would come second in the process and would follow on unequal treatment as a *post-hoc* legitimization for this unequal treatment. Supporting this sequence, Mantler et al. (2003, p. 150) found that in their studies, “the attribution sequence became increasingly influenced by personal biases and social attitudes.” This aligns with the finding that the mediation models reported here work equally well when mediator and dependent variable are switched.

In a practical sense, the above considerations suggest that those conforming to a moral norm might either first stigmatize and then discriminate against those deviating from the moral, or discriminate first and then stigmatize those deviating from a moral norm. For those being excluded or discriminated against, order will arguably matter little. Rather, the question of causality might be most relevant in terms of identifying the most promising routes for interventions. In terms of interventions, being aware that the moralization process can unfold both ways is valuable: It suggests that both attenuating accounts of responsibility for poor health outcomes in public discourse is relevant, and discouraging exclusion, discrimination, and different treatment is relevant. To the extent that health is presented in moral terms – as has been demonstrated repeatedly (e.g., Weiner et al., 1988; Weiner, 1995; Rozin, 1999; Townend, 2009) – it is made blameworthy purely by association of one stage with the other. Morality implies controllability, controllability implies responsibility, and responsibility implies blameworthiness (e.g., Weiner, 1995; Mantler et al., 2003). Specifically, it seems important to investigate interventions, communication styles, and framings of persuasive messages that lower the automatic coupling of health with morality through presuppositions of responsibility and controllability (e.g., Weiner, 1995; Mantler et al., 2003). At the same time, and acknowledging the fact that people might exclude others first and only afterward engage in legitimizing cognitions such as stigmatization, exclusion and unequal treatment of people deviating from a health norm should be discouraged. Unequal treatment and exclusion of citizens not conforming to a health norm needs to be acknowledged and discouraged. This is an important task particularly in relation to governments’ and communities’ striving toward healthier populations, as the example about smoke-free inner cities below illustrates.

### Who Is Responsible for Health?

What the current research highlights is that, in their attempt to do good for their citizens, communities might worsen existing disparities. Consider the established difference in health outcomes along the lines of socio-economic status (e.g., Adler and Ostrove, 1999): The more income and the better education someone has, the healthier this person will be. While the causes of this relationship are not conclusively determined, the consequences can easily be drafted: Those who are already suffering from poor health outcomes, less healthy nutrition, and smoke in larger numbers will face exclusion, less solidarity, and calls for paying more health insurance. Proportionally, the group that will be most negatively affected by increasing health moralization are citizens with low socio-economic status. A city in the northern part of the Netherlands is striving to be the first smoke-free city of the Netherlands. While in and of itself, this sounds like a commendable goal, it also implies that disproportionally many poorer inhabitants will not be able to go into the inner city anymore. This shows that a much more nuanced approach to public health is warranted. It also underlines a further problem of health moralization: While it burdens citizens with the full responsibility for their health outcomes, it simultaneously frees governments and institutions from taking their responsibility for citizens’ health outcomes and social cohesion.

One example that might illustrate such a process can be found in the recent liberalization of the European sugar market. This liberalization will lead to higher usage of sugars in food that were previously strictly regulated within the European Union, such as isoglucose and high fructose corn syrup. Because these sugars are associated with diabetes and obesity, researchers have issued explicit warnings that the European Union “can expect to see a dramatic increase in obesity and diabetes” (Jacobsen, 2016). This example might reflect a shift of governments’ responsibility to citizens’ responsibility, where loosening the regulations for sugar aligns with the conviction that consumers are personally responsible for buying or not buying products containing the syrup. Thus, the moralized discourse about health and lifestyle potentially worsens the problems that should be tackled, by freeing policy makers and corporations from taking responsibility for the people. The current research thus points to the specific responsibility of governments, policy makers, and institutions to carefully consider their communication of norms so as not to inadvertently divide society. The Dutch case provides an example for this notion: A core feature of the proclaimed participatory society is solidarity with others (Troonrede, 2013). As the studies presented here unanimously demonstrate, perceiving health as a moral norm actually undermines exactly this goal. Rather than showing more solidarity, a divided society surfaces characterized by endorsement of unequal treatment, exclusion of others and less solidarity.

The role of institutions and governments in communicating has been documented by prior research. For instance, Latner et al. (2007) showed that the media, television, and written press contribute to developing and maintaining stigmatizing attitudes toward obese children. Relatedly, Rich and Evans (2005) criticize the social construction and public representation of certain facts and knowledge about obesity, while “on closer inspection few such certainties are to be found” (p. 341). Thus, public discourse has been shown to create concern and “moral panic” regarding obesity (Flint et al., 2016), which feeds into stigmatization and discrimination of obese children and adults. The current research shows that obesity is certainly not the only health-related domain affected by such discourse, but that other domains are equally affected. Worryingly, such domains can be quite vague, such as when a multi-faceted construct as lifestyle is concerned, or downright unfair, such as when sick people are concerned. Health moralization might elicit an overly exclusive lens pushing those who conform into condemning everyone who appears to defy the norm. This would suggest that the impact of moralized discourse and persuasion is more severe and wide-spread than thought, with stigmatization and discrimination then affecting many more people in society besides overweight people.

### Limitations and Routes for Future Research

In light of the above considerations, a promising route for future research concerns systematic investigations into the antecedents of moralization. Prior research demonstrated that people can construe almost any issue in moral and in non-moral terms when asked to do so (Van Bavel et al., 2012), and that people differ regarding which issues they construe in moral or non-moral terms (Wright et al., 2008). However, to the best of my knowledge, systematic insights into how and why people end up seeing some issues through a moral lens and others as preferential are missing. Indeed, Skitka (personal communication with the author), deemed the question of antecedents of moral convictions “the million-dollar question.” In the meantime, some research has shown that moral emotions such as disgust can elicit moralization (Wisneski and Skitka, 2017), thereby supporting Rozin’s (1999) proposition that moralization comes about through both cognitive and emotional routes. Note that, similar to the above discussion of decision-stage models of attribution, also here it appears that moralization elicits disgust just as disgust can elicit moralization. Since the controllability dimension plays an essential role in eliciting moral emotions such as disgust, contempt, and outrage (Rudolph and Tscharaktschiew, 2014), it appears to provide a potent lever to prevent stigmatization and the unwarranted impairment of social cohesion both through a cognitive and an affective route.

While experimental research designs would valuably complement the survey studies reported here, future research should also engage in longitudinal and cross-cultural research. I have based my theoretical reasoning on plausible assumptions about the increasing moralization of health, which are supported by prior research (e.g., Rozin, 1999; Townend, 2009). However, whether moralization really is increasing and at which rate and what differences exist between cultures regarding health moralization cannot be addressed based on the research designs employed in the current research. I have shown that among diverse samples from different national and age backgrounds, the prevalence of moralization was about 20 percent. Thus, one fifth of respondents seemed to moralize health at high levels. First, it seems rewarding to examine what differentiates the 20 percent of high moralizers from the rest. Why do some people moralize health and lifestyle while others feel no urge to do so? Obviously, this question ties in with the search into antecedents of moralization noted above. The roughly 20 percent of high moralizers also suggests that the effects we have seen in the studies reported here are not mere similarity vs. dissimilarity effects where people discriminate against others who are different from themselves. Future research might want to investigate whether these prevalence rates replicate across diverse nations and cultures, but might also use these experimentally. In a sample that is repeatedly exposed to moralizing messages about health, the proportion of high moralizers should increase over time. By the same logic, the proportion of high moralizers might decrease after repeated exposure to non-moralizing messages about health, thereby probably identifying valuable routes for interventions.

Second, the question of causality might be addressed in a more sophisticated way with longitudinal studies. It appears plausible to assume that in contexts or cultures where health moralization is still in its infancy, those conforming to the health norm will go through the decision-stage models in the order reported here. However, when health moralization is more widespread and prevalent, the order might change and follow the reverse path (as displayed in Appendix B), because personal biases guide social judgment (e.g., Alicke, 2000; Mantler et al., 2003). Finally, the current research might be valuably complemented by research going beyond to self-reports and behavioral intentions. Studies considering more objective measures and real behavior, for instance, by measuring distance when sitting next to someone deviating from a moral health-related norm (Haidt et al., 2003; Wright et al., 2008), or cooperativeness and ability to resolve conflicts in groups consisting of people conforming to or deviating from a moral health-related norm (Skitka et al., 2005) would be relevant next steps. Below, I highlight another intriguing follow-up question concerning subjective construals of health and lifestyle.

### Subjective Construals of Health and Lifestyle

Complementing and extending prior research, the current research showed that own health and lifestyle are relevant for predicting stigmatization and important indicators of social cohesion. The studies reported here relied on self-reported, more or less objective conformity to a health norm such as non-smoking, being of normal weight, or having a healthy lifestyle. However, these self-reports also give rise to a number of intriguing questions. The most obvious one concerns the question whether the perception that one’s health is good and one’s own lifestyle is healthy is indeed grounded in reality. Does the presumed consensus about what is healthy actually find reflection in normal people’s construal of health? Further, particularly lifestyle involves many different aspects, some of which people might weigh heavier than others. Thus, which of these aspects do people rely on when deciding whether or not their lifestyle is healthy? Lifestyle is a construct with many different aspects concerning exercise, nutrition, sleep, alcohol consumption, and so on. Surely, most people do better on some of these aspects than on others. Thus, which weight do people give to those aspects when considering their own, but also others, lifestyles? If indeed people use their own lifestyle as a basis for moralization, thus tend to moralize those attributes they fulfill themselves (Pinker, 2008), then there should be great variation in aspects of lifestyle used to stigmatize others. And, related to the previous question, how would people evaluate others who conform to some but deviate from other aspects of a healthy lifestyle? What about the overweight person who exercises three times a week and is a vegetarian? What about the smoker who never drinks alcohol? What about the normal weight person who drinks excessively and eats poorly? With the last description, the typical person that comes to mind is a young adult, possibly a student or young professional, and this summarizes a big problem of health and lifestyle moralization: Many people do nothing much for certain aspects of their lifestyle. Young people for instance are on average slimmer and more healthy than older adults, despite their well-known excessive alcohol consume and poor eating habits during university. Thus, while their health outcomes might be fine, their efforts into being healthy might be negligible. This illustrates a problem inherent to moralization, namely that often outcomes rather than effort provide the foundation for moral judgment: People perceiving others through a moral lens will rarely consider how many attempts to losing weight or to quit smoking the other person has made.

## Conclusion

In providing first evidence for the negative consequences flowing from health moralization, the present research shows that governments’ good intentions in persuading entire populations to live more healthily might come at a substantial cost, especially when the discourse highlights morality. The moralization of health and lifestyle undermines social cohesion by creating a divide between those conforming to and those deviating from the health norm. The current research complemented and extended prior research by showing negative consequences of health moralization on a variety of aspects (weight, smoking, lifestyle) and on a societal level. At the same time, I have outlined a number of promising routes for follow-up research that might tackle some limitations of the studies presented here and that can address important questions revolving around the impact of health moralization at the individual, organizational, and societal level. One rather urgent route for future research concerns the consequences of health moralization on decision making among policy makers. The conviction that health is a personal choice and is under citizens’ control, as implied by its moralization, might free policy makers and institutions from their responsibility toward citizens. They might arrive at conclusions and make decisions that ultimately affect citizens negatively, thereby undermining social cohesion and the achievement of important societal goals.

Admittedly, the implications of the present research sound pessimistic. However, the insights provided here also lend themselves for deriving some recommendations at the levels of institutions, employers, and individual citizens. Firstly, communicate about health and lifestyle in an inclusive way that embraces gradations rather than either-or judgments. In other words, avoid depicting health and lifestyle as something absolute, and prefer to depict it as something people can gradually get closer to. Secondly, avoid framing health and lifestyle in terms of harm inflicted on others, and specifically avoid stressing zero-sum conflicts as these are strongly associated with moral exclusion (Opotow, 1990). Third, be aware of your own prejudice and the foundations of your moral convictions. Consider how much effort you have put into conforming to the norm of living healthily. Also consider how much effort others have put into living healthily, and try not to confuse outcome with effort.

## Ethics Statement

This study was carried out in accordance with the recommendations of the Ethical Commission of the Behavioral Research Lab of the Faculty of Economics and Business (University of Groningen) with written informed consent from all subjects. All subjects gave written informed consent in accordance with the Declaration of Helsinki. The protocol was approved by the Ethical Commission of the Behavioral Research Lab.

## Author Contributions

ST: I have single-authored the submitted manuscript, and have designed the research, acquired, analyzed, and interpreted the data. I have written the manuscript and am accountable for all aspects of the work.

## Conflict of Interest Statement

The author declares that the research was conducted in the absence of any commercial or financial relationships that could be construed as a potential conflict of interest.

## References

[B1] Adler N. E. Ostrove J. M. (1999). Socioeconomic status and health: what we know and what we don’t. *Ann. N. Y. Acad. Sci.* 896 3–15. 10.1111/j.1749-6632.1999.tb08101.x 10681884

[B2] Alicke M. D. (2000). Culpable control and the psychology of blame. *Psychol. Bull.* 126 556–574. 10.1037/0033-2909.126.4.556 10900996

[B3] Bisogni C. A. Connors M. Devine C. M. Sobal J. (2002). Who we are and how we eat: a qualitative study of identities in food choice. *J. Nutr. Educ. Behav.* 34 128–139. 10.1016/S1499-4046(06)60082-1 12047837

[B4] Crandall C. S. (1994). Prejudice against fat people: ideology and self-interest. *J. Pers. Soc. Psychol.* 66 882–894. 10.1037/0022-3514.66.5.882 8014833

[B5] De Waal F. B. (1996). *Good Natured (No. 87).* Cambridge, MA: Harvard University Press.

[B6] Ellemers N. Pagliaro S. Barreto M. Leach C. W. (2008). Is it better to be moral than smart? The effects of morality and competence norms on the decision to work at group status improvement. *J. Pers. Soc. Psychol.* 95 1397–1410. 10.1037/a0012628 19025291

[B7] Ellemers N. van den Bos K. (2012). Morality in groups: on the social-regulatory functions of right and wrong. *Soc. Pers. Psychol. Compass* 6 878–889. 10.1111/spc3.12001

[B8] Flint S. W. Čadek M. Codreanu S. C. Ivić V. Zomer C. Gomoiu A. (2016). Obesity discrimination in the recruitment process: “You’re Not Hired!”. *Frontiers in Psychology* 7:647. 10.3389/fpsyg.2016.00647. 27199869 PMC4853419

[B9] Flint S. W. Snook J. (2014). Obesity and discrimination: The next ‘big issue’? *Int. J. Discrimin. Law* 14 183–193. 10.1177/1358229114534550

[B10] Fransen M. L. Smit E. G. Verlegh P. W. (2015). Strategies and motives for resistance to persuasion: an integrative framework. *Front. Psychol.* 6:1201. 10.3389/fpsyg.2015.01201 26322006 PMC4536373

[B11] Gaertner S. L. Dovidio J. F. Anastasio P. A. Bachman B. A. Rust M. C. (1993). The common ingroup identity model: recategorization and the reduction of intergroup bias. *Eur. Rev. Soc. Psychol.* 4 1–26. 10.1080/14792779343000004 9247370

[B12] Gantman A. P. Van Bavel J. J. (2014). The moral pop-out effect: enhanced perceptual awareness of morally relevant stimuli. *Cognition* 132 22–29. 10.1016/j.cognition.2014.02.007 24747444

[B13] Gantman A. P. Van Bavel J. J. (2015). Moral perception. *Trends Cogn. Sci.* 19 631–633. 10.1016/j.tics.2015.08.004 26440123

[B14] Goh J. X. Hall J. A. Rosenthal R. (2016). Mini meta-analysis of your own studies: some arguments on why and a primer on how. *Soc. Pers. Psychol. Compass* 10 535–549. 10.1111/spc3.12267

[B15] Haidt J. (2001). The emotional dog and its rational tail: a social intuitionist approach to moral judgment. *Psychol. Rev.* 108 814–834. 10.1037/0033-295X.108.4.814 11699120

[B16] Haidt J. Koller S. H. Dias M. G. (1993). Affect, culture, and morality, or is it wrong to eat your dog? *J. Pers. Soc. Psychol.* 65 613–628. 10.1037/0022-3514.65.4.613 8229648

[B17] Haidt J. Rosenberg E. Hom H. (2003). Differentiating diversities: moral diversity is not like other kinds. *J. Appl. Soc. Psychol.* 33 1–36. 10.1111/j.1559-1816.2003.tb02071.x

[B18] Haselton M. G. Buss D. M. (2000). Error management theory: a new perspective on biases in cross-sex mind reading. *J. Pers. Soc. Psychol.* 78 81–91. 10.1037/0022-3514.78.1.81 10653507

[B19] Hayes A. F. (2012). *PROCESS: A Versatile Computational Tool for Observed Variable Mediation, Moderation, and Conditional Process Modeling [White paper].* Available at: http://www.afhayes.com/public/process2012.pdf

[B20] Heider F. (1958). *The Psychology of Interpersonal Relations.* New York, NY: John Wiley & Sons, 332.

[B21] Hollenbeck C. R. Kaikati A. M. (2012). Consumers’ use of brands to reflect their actual and ideal selves on Facebook. *Int. J. Res. Mark.* 29 395–405. 10.1016/j.ijresmar.2012.06.002

[B22] Jacobsen H. (2016). *Obesity Researcher: The EU’s New Sugar Quotas will Increase Diabetes Rates.* Available at: https://www.euractiv.com/section/agriculture-food/news/obesity-researcher-says-the-eu-s-new-sugar-quotas-will-increase-diabetes-rates/

[B23] Kesseler R. C. Mickelson K. D. Williams D. R. (1999). The prevalence, distribution, and mental health correlates of perceived discrimination in the United States. *J. Health Soc. Behav.* 40 208–230. 10.2307/2676349 10513145

[B24] Knowles E. S. Linn J. A. (eds) (2004). *Resistance and Persuasion.* Mahwah, NJ: Psychology Press.

[B25] Latner J. D. Rosewall J. K. Simmonds M. B. (2007). Childhood obesity stigma: association with television, videogame, and magazine exposure. *Body Image* 4 147–155. 10.1016/j.bodyim.2007.03.002 18089260

[B26] Link B. G. Phelan J. C. (2001). Conceptualizing stigma. *Annu. Rev. Soc.* 27 363–385. 10.1146/annurev.soc.27.1.363

[B27] Mantler J. Schellenberg E. G. Page J. S. (2003). Attributions for serious illness: are controllability, responsibility and blame different constructs? *Can. J. Behav.* 35 142–152. 10.1037/h0087196

[B28] Morrison M. A. Morrison T. G. Pope G. A. Zumbo B. D. (1999). An investigation of measures of modern and old-fashion sexism. *Soc. Indic. Res.* 48 39–50. 10.1023/A:1006873203349

[B29] Mulder L. B. (2008). The difference between punishments and rewards in fostering moral concerns in social decision making. *J. Exp. Soc. Psychol.* 44 1436–1443. 10.1016/j.jesp.2008.06.004

[B30] OECD (2011). *Perspectives on Global Development 2012: Social Cohesion in a Shifting World.* Paris: OECD Publishing

[B31] Olson J. M. Zanna M. P. (1993). Attitudes and attitude change. *Annu. Rev. Psychol.* 44 117–154. 10.1146/annurev.ps.44.020193.001001

[B32] Opotow S. (1990). Moral exclusion and injustice: an introduction. *J. Soc. Issues* 46 1–20. 10.1111/j.1540-4560.1990.tb00268.x

[B33] Petty R. E. Cacioppo J. T. (1986). The elaboration likelihood model of persuasion. *Adv. Exp. Soc. Psychol.* 19 123–205. 10.1016/S0065-2601(08)60214-2

[B34] Pinker S. (2008). *The Moral Instinct.* Available at: http://www.nytimes.com/2008/01/13/magazine/13Psychology-t.html

[B35] Puhl R. Brownell K. D. (2001). Bias, discrimination, and obesity. *Obesity* 9 788–805. 10.1038/oby.2001.108 11743063

[B36] Puhl R. M. Luedicke J. (2012). Weight-based victimization among adolescents in the school setting: Emotional reactions and coping behaviors. *J. Youth Adolesc.* 41 27–40. 10.1007/s10964-011-9713-z 21918904

[B37] Rich E. Evans J. (2005). ‘Fat ethics’–The obesity discourse and body politics. *Soc. Theory Health* 3 341–358. 10.1057/palgrave.sth.8700057

[B38] Rozin P. (1999). The process of moralization. *Psychol. Sci.* 10 218–221. 10.1111/1467-9280.00139

[B39] Rozin P. Markwith M. Stoess C. (1997). Moralization and becoming a vegetarian: the transformation of preferences into values and the recruitment of disgust. *Psychol. Sci.* 8 67–73. 10.1111/j.1467-9280.1997.tb00685.x

[B40] Rozin P. Singh L. (1999). The moralization of cigarette smoking in the United States. *J. Consum. Psychol.* 8 321–337. 10.1207/s15327663jcp0803_07

[B41] Rudolph U. Tscharaktschiew N. (2014). An attributional analysis of moral emotions: naïve scientists and everyday judges. *Emot. Rev.* 6 344–352. 10.1177/1754073914534507

[B42] Sagarin B. J. Cialdini R. B. Rice W. E. Serna S. B. (2002). Dispelling the illusion of invulnerability: the motivations and mechanisms of resistance to persuasion. *J. Pers. Soc. Psychol.* 83 526. 10.1037/0022-3514.83.3.526 12219852

[B43] Schubert T. W. Otten S. (2002). Overlap of self, ingroup, and outgroup: pictorial measures of self-categorization. *Self Identity* 1 353–376. 10.1080/152988602760328012

[B44] Shaver K. G. (1985). *The Attribution of Blame: Causality, Responsibility, and Blameworthiness.* New York, NY: Springer-Verlag.

[B45] Sikorski C. Luppa M. Angermeyer M. C. Schomerus G. Link B. Riedel-Heller S. G. (2015). The association of BMI and social distance towards obese individuals is mediated by sympathy and understanding. *Soc. Sci. Med.* 128 25–30. 10.1016/j.socscimed.2015.01.002 25577288

[B46] Sikorski C. Luppa M. Brähler E. König H. H. Riedel-Heller S. G. (2012). Obese children, adults and senior citizens in the eyes of the general public: results of a representative study on stigma and causation of obesity. *PLoS One* 7:e46924. 10.1371/journal.pone.0046924 23071664 PMC3470564

[B47] Skitka L. J. (2010). The psychology of moral conviction. *Soc. Pers. Psychol. Compass* 4 267–281. 10.1111/j.1751-9004.2010.00254.x

[B48] Skitka L. J. Bauman C. W. Sargis E. G. (2005). Moral conviction: another contributor to attitude strength or something more? *J. Pers. Soc. Psychol.* 88 895–917. 10.1037/0022-3514.88.6.895 15982112

[B49] Skitka L. J. Mullen E. (2002). Understanding judgments of fairness in a real-world political context: a test of the value protection model of justice reasoning. *Pers. Soc. Psychol. Bull.* 28 1419–1429. 10.1177/014616702236873

[B50] Skitka L. J. Washburn A. N. Carsel T. S. (2015). The psychological foundations and consequences of moral conviction. *Curr. Opin. Psychol.* 6 41–44. 10.1016/j.copsyc.2015.03.025

[B51] Skowronski J. J. Carlston D. E. (1987). Social judgment and social memory: the role of cue diagnosticity in negativity, positivity, and extremity biases. *J. Pers. Soc. Psychol.* 52 689–699. 10.1037/0022-3514.52.4.689

[B52] Skowronski J. J. Carlston D. E. (1989). Negativity and extremity biases in impression formation: a review of explanations. *Psychol. Bull.* 105 131–142. 10.1037/0033-2909.105.1.131

[B53] Skowronski J. J. Carlston D. E. (1992). Caught in the act: when impressions based on highly diagnostic behaviours are resistant to contradiction. *Eur. J. Soc. Psychol.* 22 435–452. 10.1002/ejsp.2420220503

[B54] Täuber S. van Zomeren M. Kutlaca M. (2015). Should the moral core of climate issues be emphasized or downplayed in public discourse? Three ways to successfully manage the double-edged sword of moral communication. *Clim. Change* 130 453–464. 10.1007/s10584-014-1200-6

[B55] Townend L. (2009). The moralizing of obesity: A new name for an old sin? *Crit. Soc. Policy* 29 171–190. 10.1177/0261018308101625

[B56] Troonrede (2013). Available at: https://www.rijksoverheid.nl/documenten/toespraken/2013/09/17/troonrede-2013

[B57] Van Bavel J. J. Packer D. J. Haas I. J. Cunningham W. A. (2012). The importance of moral construal: moral versus non-moral construal elicits faster, more extreme, universal evaluations of the same actions. *PLoS One* 7:e48693. 10.1371/journal.pone.0048693 23209557 PMC3509100

[B58] van Leeuwen F. Park J. H. Penton-Voak I. S. (2012). Another fundamental social category? Spontaneous categorization of people who uphold or violate moral norms. *J. Exp. Soc. Psychol.* 48 1385–1388. 10.1016/j.jesp.2012.06.004

[B59] van Zomeren M. Postmes T. Spears R. (2012). On conviction’s collective consequences: integrating moral conviction with the social identity model of collective action. *Br. J. Soc. Psychol.* 51 52–71. 10.1111/j.2044-8309.2010.02000.x 22435846

[B60] van Zomeren M. Postmes T. Spears R. Bettache K. (2011). Can moral convictions motivate the advantaged to challenge social inequality? Extending the social identity model of collective action. *Group Process. Intergroup Relat.* 14 735–753. 10.1177/1368430210395637

[B61] Verschoor W. (2015). De participatiesamenleving is niet van vandaag of gisteren [The participation society is not of today or yesterday]. *Podium Bioethiek* 22 8–10.

[B62] Weiner B. (1986). “Attribution, emotion, and action,” in *Handbook of Motivation and Cognition: Foundations of Social Behavior*, eds Sorrentino R. M. Higgins E. T. (New York, NY: Guilford), 281–312.

[B63] Weiner B. (1995). *Judgments of Responsibility: A Foundation for a Theory of Social Conduct.* New York, NY: Guilford Press.

[B64] Weiner B. Perry R. P. Magnusson J. (1988). An attributional analysis of reactions to stigmas. *J. Pers. Soc. Psychol.* 55 738–748. 10.1037/0022-3514.55.5.738 2974883

[B65] White K. Dahl D. W. (2007). Are all out-groups created equal? Consumer identity and dissociative influence. *J. Consum. Res.* 34 525–536. 10.1086/520077

[B66] Wisneski D. C. Skitka L. J. (2017). Moralization through moral shock: exploring emotional antecedents to moral conviction. *Pers. Soc. Psychol. Bull.* 43 139–150. 10.1177/0146167216676479 27872393

[B67] Wojciszke B. (1994). Multiple meanings of behavior: construing actions in terms of competence or morality. *J. Pers. Soc. Psychol.* 67 222–232. 10.1037/0022-3514.67.2.222

[B68] Wojciszke B. (2005). Morality and competence in person-and self-perception. *Eur. Rev. Soc. Psychol.* 16 155–188. 10.1080/10463280500229619

[B69] Wood W. (2000). Attitude change: Persuasion and social influence. *Annu. Rev. Psychol.* 51 539–570. 10.1146/annurev.psych.51.1.539 10751980

[B70] Wright J. Cullum J. Schwab N. (2008). The cognitive and affective dimensions of moral conviction: implications for attitudinal and behavioral measures of interpersonal tolerance. *Pers. Soc. Psychol. Bull.* 34 1461–1476. 10.1177/0146167208322557 18685130

[B71] Wright J. C. Grandjean P. T. McWhite C. B. (2013). The meta-ethical grounding of our moral beliefs: evidence for meta-ethical pluralism. *Philos. Psychol.* 26 336–361. 10.1080/09515089.2011.633751

