# Supplementary material for: Moralized Health-Related Persuasion Undermines Social Cohesion
Source: Front Psychol. 2018 Jun 12;9:909. doi: 10.3389/fpsyg.2018.00909 (PMC6005884; doi:10.3389/fpsyg.2018.00909)
Supplement: Supplementary file 1 [file Presentation_1.PDF]

APPENDIX A: Overview over measures

**Predictor: Moralization**	
Study 1	Living healthily is a moral obligation.
	Citizens have a duty to do everything to stay healthy.
	People are not obliged to live healthily. (reverse coded)
Study 2a	It is a moral duty to follow a healthy lifestyle.
	People with an unhealthy lifestyle are a burden for society.
Study 2b	It is a moral duty to follow a healthy lifestyle.
	People with an unhealthy lifestyle are a burden for society.
Study 3	Having an unhealthy lifestyle …
	…is not a social thing to do,
	…is something I do object to,
	…is something I morally disapprove of
	…is morally incorrect

Note. All items were measured on 5-point Likert scales from 1 (strongly disagree), 2 (somewhat disagree), 3 (neither disagree nor agree), 4 (somewhat agree) to 5 (strongly agree).

**Mediator: Stigmatization**	
Study 1	I really don't like smokers much.<br>I don't have many friends who smoke.<br>I tend to think that people who smoke are a little untrustworthy.<br>Although some smokers are surely smart, in general, I think they tend not to be quite as bright as non-smokers.<br>I have had a hard time taking smokers too seriously.<br>Smokers make me feel somewhat uncomfortable.<br>If I were an employer looking to hire, I might avoid hiring a smoker.
Study 2a	Smokers are lazy.<br>Smokers lack self-control.<br>Smokers don’t try hard enough to stop.
Study 2b	People with overweight are lazy.<br>People with overweight lack self-control.<br>People with overweight don’t try hard enough to lose weight.
Study 3	I dislike colleagues with an unhealthy lifestyle.<br>I think that colleagues with an unhealthy lifestyle are unreliable.<br>In general, I think that colleagues with an unhealthy lifestyle are less intelligent.<br>I have hard times taking colleagues with an unhealthy lifestyle serious.<br>Colleagues with an unhealthy lifestyle make me feel uncomfortable.<br>If I would be an employer I would not hire an applicant with an unhealthy lifestyle.<br>I do not want to have contact with a colleague with an unhealthy lifestyle.

Note. All items were measured on 5-point Likert scales from 1 (strongly disagree), 2 (somewhat disagree), 3 (neither disagree nor agree), 4 (somewhat agree) to 5 (strongly agree).

**Dependent variable: Social Cohesion**	
Study 1	Please choose the number of the graphic that best represents the extent to which you think that norms and values of the following groups overlap: **smokers** and **non-smokers** - **healthy** and **ill people** - **smokers** and **society - non-smokers** and **society** - **healthy people** and **society** - **ill people** and **society**<br>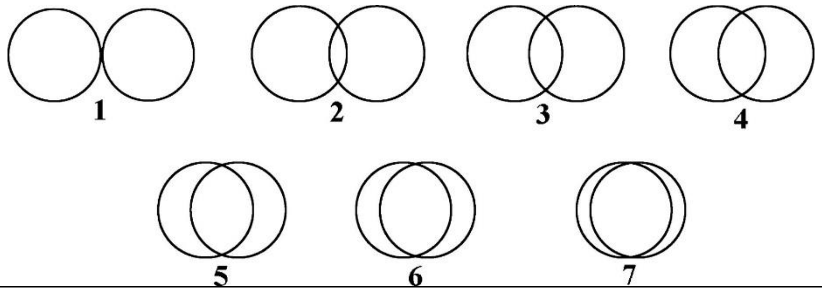
Study 2a and 2b	*Showing solidarity*<br>I would be willing to pay more health insurance premium ...<br>... to show solidarity for people who are sick.<br>... to show solidarity for people with an unhealthy lifestyle.<br>... to show solidarity for people who smoke.<br>... to show solidarity for people who have an unhealthy weight.<br>*Expectations of solidarity*<br>I am convinced that other people would help me if I am sick.<br>I believe that others would support me if I am sick.<br>I expect people to show solidarity when I am sick.<br>*Who should pay more?*<br>Imagine that the health insurance premium goes up. Who should pay more in your opinion?<br>People who are ill (1) – People who are healthy (7)<br>Smokers (1) – Non-smokers (7)<br>People who are overweight (1) – People who have normal weight (7)<br>People who are living unhealthily (1) - People who are living healthily (7)

Note. Unless indicated otherwise, all items were measured on 5-point Likert scales from 1 (strongly disagree), 2 (somewhat disagree), 3 (neither disagree nor agree), 4 (somewhat agree) to 5 (strongly agree), with the exception of the IOS scale and the items referring to who should pay more.

**Dependent variable: Social Cohesion (continued)**	
Study 3	*Categorization*
	Independently of their lifestyle, me and my colleagues form one team.
	Although me and my colleagues have a distinct lifestyle, we all pull together on one string.
	At work, I have the feeling that colleagues with a healthy lifestyle and colleagues with an unhealthy lifestyle belong to two different groups. (recoded)
	Colleagues with a healthy lifestyle work together well with colleagues with an unhealthy lifestyle.
	Exclusion
	Employees with an unhealthy lifestyle are excluded.
	Employees with an unhealthy lifestyle are ignored.
	Others point out the lacking intelligence of employees with an unhealthy lifestyle.
	Discrimination
	Colleagues with an unhealthy lifestyle often do not receive the opportunity for a good job.
	Colleagues with an unhealthy lifestyle are treated unfair at the workplace.
	Colleagues with an unhealthy lifestyle are treated unkindly.
	Colleagues with an unhealthy lifestyle are treated with less respect.
	Colleagues with an unhealthy lifestyle are insulted at the workplace.

Note. Unless indicated otherwise, all items were measured on 5-point Likert scales from 1 (strongly disagree), 2 (somewhat disagree), 3 (neither disagree nor agree), 4 (somewhat agree) to 5 (strongly agree).
