# Supplementary material for: Moralized Health-Related Persuasion Undermines Social Cohesion
Source: Front Psychol. 2018 Jun 12;9:909. doi: 10.3389/fpsyg.2018.00909 (PMC6005884; doi:10.3389/fpsyg.2018.00909)
Supplement: Supplementary file 2 [file Presentation_2.PDF]

Appendix B

Supplementary analyses

The supplementary analyses provide a more detailed overview of descriptive statistics and results of the simple mediation analyses per construct per study. At the end of the supplementary analysis, power analyses per study are reported.

Study 1

In Study 1, the social cohesion indicator inclusion was measured by means of six IOS items. Factor analysis showed that these items loaded on two factors, inclusion of deviants, which has been reported in the main analyses, and inclusion of conformers. This latter factor was analyzed using the same procedure as described in the main analyses (simple mediations with z-standardized variables using model 4 with 5000 re-samples as pre-defined, PROCESS macro, Hayes, 2012). The analysis yielded a significant mediator model, $F(1,230) = 10.40$, $p = .001$, $R^2 = 4.33\%$ showing that the more non-smokers saw health as a moral obligation, the stronger the anti-smoker attitudes they reported (β = .21, $SE = 0.06$, $t = 3.22$, $p = .001$). However, not surprisingly, neither anti-smoker attitudes (β = .05, $SE = 0.07$, $t = 0.70$, $p = .486$) nor health moralization (β = .06, $SE = 0.07$, $t = 0.89$, $p = .373$) predicted the inclusion of those conforming to the health norm. Accordingly, both the direct ($CI_{95\%}$ [-.071, .193]) and the indirect effect ($CI_{95\%}$ [-.014, .042]) of health moralization on inclusion of those conforming to the health norm were non-reliable.

Study 2a

In Study 2a, social cohesion was measured through the indicators solidarity and equal treatment in terms of paying for health insurance. The main analyses showed that respondents' willingness to show solidarity with those deviating from the health norm decreased as a function of their moralization of health. An interesting question pertains to whether this finding extended to all groups of deviants or only to some groups. To examine this, *t*-tests were performed. These showed that willingness to show solidarity was significantly below the mid-point of the scale for all groups, all $t(765) > 2.90$, all $p < .004$. When examining respondents' endorsement of unequal payment, a similar pattern emerged. These showed that unequal payment was endorsed for all groups, all $t(765) > 33.00$, all $p < .001$. This suggests that conformers with a health norm subsume people who are sick with diverse groups such as smokers, overweight people, and people with unhealthy lifestyles. Given that sickness can be caused by many different factors, many of these outside of people's control, this suggests an overly inclusive categorization of groups under the category "deviants".

Analyzing these constructs using the same procedure as described in the main analyses (simple mediations with z-standardized variables using model 4 with 5000 re-samples as pre-defined, PROCESS macro, Hayes, 2012), yielded a significant mediator model, $F(1,764)=176.94$, $p<.001$, $R^2=18.80\%$, showing that the more non-smokers saw health as a moral obligation, the stronger the anti-smoker attitudes they reported (β = .45, $SE = 0.03$, $t = 13.99$, $p < .001$). Table $1_{\text{Supplement}}$ provides an overview over the dependent variable models per construct: Anti-smoker attitudes were associated with less willingness to show solidarity with diverse groups deviating from the health norm (i.e., ill people, overweight people, smokers, and people with an unhealthy lifestyle).

Health moralization still explained a significant amount of variance besides anti-smoker attitudes. Nevertheless, the indirect effect of health moralization on willingness to show solidarity with others through anti-smoker attitudes was significant and reliable (Z= -5.23, p<.001 [$CI_{95\%}$ -.136, -.054]). The dependent variable model for non-smokers' expectations of solidarity was not significant. Finally, non-smokers' opinion that those deviating from the health norm in society should pay more health insurance was strongly affected by both anti-smoker attitudes and by health moralization. The indirect effect was significant and reliable (Z= 5.45, p<.001 [$CI_{95\%}$ .059, .133]).

Study 2b

Just as in Study 2a, respondents' willingness to show solidarity was significantly below the midpoint of the scale for all groups, all $t(446) > 2.30$, all $p < .02$. The same pattern emerged for respondents' endorsement of unequal payment, all $t(446) > 24.00$, all $p < .001$. Again, this suggests an overly inclusive categorization of others as deviants, given that sickness can be caused by many factors outside of people's control. In Study 2b, social cohesion was measured through the indicators solidarity and equal treatment in terms of paying for health insurance. Analyzing these constructs using the same procedure as described in the main analyses (simple mediations with z-standardized variables using model 4 with 5000 re-samples as pre-defined, PROCESS macro, Hayes, 2012), yielded a significant mediator model, $F(1,445)=76.68$, $p<.001$, $R^2=14.70\%$, showing that the more normal weight respondents saw health as a moral obligation, the stronger the anti-fat attitudes they reported ($\beta = .36$, $SE = 0.04$, $t = 8.76$, $p < .001$). Table $2_{\text{Supplement}}$ provides an overview over the dependent variable models per construct: In turn, anti-fat attitudes were associated with marginally less willingness to show solidarity with diverse groups deviating from the health norm (i.e., ill people, overweight people, smokers, and people with an unhealthy lifestyle). Health moralization still explained a significant amount of variance besides anti-fat attitudes. Different from the non-smokers sample, while the direct effect of moralization on willingness to show solidarity was significant and reliable, the indirect effect of health moralization on willingness to be solidary with others through anti-fat attitudes was only marginally significant (Z= -1.90, p=.058) and not reliable ($CI_{95\%}$ -.077, .003). Different from the non-smokers sample, normal weight respondents' expectations of solidarity from others in society were significantly affected both by anti-fat attitudes and by health moralization. Specifically, the more respondents moralized health, the more solidarity they expected from others in society in case they would need help. By contrast, the stronger respondents' anti-fat attitudes, the less solidarity they expected from others in society. The indirect effect was significant and reliable (Z= -2.77, p = .006 [$CI_{95\%}$ -.096, -.017]). Finally, normal weight respondents' opinion that those groups in society who deviate from the health norm should pay more health insurance strongly increased with both anti-fat attitudes and health moralization. The indirect effect was significant and reliable (Z= 3.09, p = .002 [$CI_{95\%}$ .018, .111]).

Study 3

In Study 3, social cohesion was measured through the indicators inclusion and equal treatment in terms of non-discrimination and non-exclusion. Analyzing these constructs using the same procedure as described in the main analyses (simple mediations with z-standardized variables using model 4 with 5000 re-samples as pre-defined, PROCESS macro, Hayes, 2012), yielded a significant mediator model, $F(1,120)=10.73$, $p<.01$, $R^2=8.21\%$, showing that the more respondents

with healthy lifestyles saw lifestyle as a moral obligation, the more negative attitudes they reported towards colleagues with unhealthy lifestyles ($\beta = .31$, $SE = 0.09$, $t = 3.28$, $p < .01$). Table $3_{Supplement}$ provides an overview over the dependent variable models per construct: Stigmatization was associated with significantly lower categorization of colleagues with unhealthy lifestyles as forming one group with the respondents. The indirect effect was significant and reliable, $Z= -2.88$, $p = .004$ ($CI_{95\%}$ -.262, -.058). Lifestyle moralization was further associated with significantly lower levels of non-inclusion. The direct effect was significant. The indirect effect through stigmatization was significant and reliable, $Z= -2.76$, $p = .006$ ($CI_{95\%}$ -.254, -.047). Finally, lifestyle moralization was associated with significantly lower levels of equal treatment as reflected by non-discrimination. The indirect effect through stigmatization was significant and reliable, $Z= -2.98$, $p = .003$ ($CI_{95\%}$ -.311, -.081).

Note. For Study 3, recruitment efforts resulted in only 75 respondents first. Due to concerns about being underpowered given the analyses presented, more participants were recruited for Study 3 after the first round of reviews, resulting in the 123 respondents reported here. Oneway ANOVAs revealed no difference between first and second recruitment in the constructs of interest, all *F*'s < 0.13, all *p*'s > .720.

Note. Bonferroni corrections for repeated analyses suggest a cut-off point of alpha = .017 for Studies 2a, 2b, and 3, in order to account for alpha error accumulation due to repeated analyses. The significant indirect effects observed in Studies 2a, 2b, and 3 all fell well below this cut-off point (al $p < .007$).

Note. The mediation models reported in the main analyses also work when the mediator and dependent variables are switched. Table $4_{Supplement}$ provides an overview over the statistics for the mediator model across studies, showing that in all studies, health moralization was associated with significantly lower social cohesion (mediator). Table $5_{Supplement}$ shows the corresponding dependent variable model, where the effect of health moralization on stigmatization mediated through social cohesion was examined. For Study 1, the indirect effect was marginally significant, $Z= 1.83$, $p = .068$ ($CI_{95\%}$ .006, .093). For all other studies, the indirect effects were highly significant, all $Z's > 3.28$, all $p's < .002$ ($CI_{95\%}$ .052, .111; $CI_{95\%}$ .020, .091; $CI_{95\%}$ .046, .222, for studies 2a, 2b, and 3, respectively). The direct effects of health moralization on stigmatization were highly significant in all studies, all $t's > 2.08$, $p's < .04$ ($CI_{95\%}$ .047, .292; $CI_{95\%}$ .301, .438; $CI_{95\%}$ .223, .390; $CI_{95\%}$ .011, .371, for studies 1, 2a, 2b, and 3, respectively). Thus, health moralization was associated with significantly higher levels of stigmatization of those deviating from a health norm, and this effect was mediated by decreased levels of social cohesion. These findings are discussed in detailed in the discussion section under the header *Some thoughts about causality*.

Post Hoc Statistical Power Analysis

In recruiting participants for the studies reported here, I was guided by the wish to increase precision by increasing statistical power. Increasing precision decreases the probability of making both Type I and Type II errors (Button et al., 2013). Increasing sample size and using reliable measurements both serve to reduce the measurement error (MacKinnon, 2013), thereby increasing precision. Thus, for all studies reported in this paper, large sample sizes were aspired

(more or less successfully) and reliable measurements have been used. Post hoc power analyses were conducted for all studies reported here using the software package G*Power 3.1 (Faul, Erdfelder, Buchner, & Lang, 2009). The sample sizes of $N = 232$ (Study 1), 766 (Study 2a), 447 (Study 2b), and 123 (Study 3) were used for the statistical power analyses and a 2-predictor variable equation was used as a baseline. The recommended effect sizes used for this assessment were as follows: small ($f2 = .02$), medium ($f2 = .15$), and large ($f2 = .35$). The alpha level used for this analysis was $p < .05$, and the effect was modelled two-tailed, given that the hypotheses were directional. The post hoc analyses revealed the statistical power for all studies was .99 for detecting small, moderate, and large effects. There was thus more than adequate power to detect the effects reported here.

Table 1 Supplement

Dependent variable models for simple mediation, Study 2a (non-smoker sample).

	DV = Willingness to show solidarity, $F(2,763)=39.42$, $p<.001$, $R^2=9.37\%$		
Predictor	b^a	*SE*	*t*
Constant	0.00	0.03	0.00
Moralization	-0.13	0.04	-3.40***
Anti-smokers attitude	-0.23	0.04	-5.94***
	DV = Expectations of solidarity, $F(2,763)=0.51$, $p=.599$, $R^2=0.26\%$		
Predictor	b^a	*SE*	*t*
Constant	0.00	0.03	0.00
Moralization	0.05	0.04	1.30
Anti-smokers attitude	-0.04	0.04	-1.09
	DV = Pay more health insurance, $F(2,763)=98.28$, $p<.001$, $R^2=20.48\%$		
Predictor	b^a	*SE*	*t*
Constant	0.00	0.03	0.00
Moralization	0.31	0.04	8.47***
Anti-smokers attitude	0.22	0.04	6.14***

Note. [a] Standardized regression coefficients; $^{*}p < .05$, $^{**}p < .01$, $^{***}p < .001$.

Table 2 Supplement

Dependent variable models for simple mediation, Study 2b (normal weight sample).

	DV = Willingness to show solidarity, $F(2,444)=18.86$, $p<.001$, $R^2=7.83\%$		
Predictor	b^a	*SE*	*t*
Constant	0.00	0.06	0.00
Moralization	-0.22	0.05	-4.63***
Anti-fat attitude	-0.10	0.05	-1.96
	DV = Expectation of solidarity, $F(2,444)=6.70$, $p<.01$, $R^2=2.93\%$		
Predictor	b^a	*SE*	*t*
Constant	0.00	0.04	0.00
Moralization	0.14	0.05	3.14**
Anti-fat attitude	-0.14	0.05	-2.94**
	DV = Pay more health insurance, $F(2,444)=46.62$, $p<.001$, $R^2=17.36\%$		
Predictor	b^a	*SE*	*t*
Constant	0.00	0.04	0.00
Moralization	0.31	0.04	7.10***
Anti-fat attitude	0.16	0.05	3.32**

Note. [a] Standardized regression coefficients; * $p < .05$, ** $p < .01$, *** $p < .001$.

Table 3 Supplement

Dependent variable models for simple mediation, Study 3 (healthy lifestyle sample).

	DV = Categorization as same group, $F(2,119)=23.51$, $p<.001$, $R^2=28.32\%$		
Predictor	b^a	*SE*	*t*
Constant	0.06	0.08	0.80
Moralization	-0.06	0.08	-0.79
Negative attitude	-0.48	0.08	-6.30***
	DV = Inclusion, $F(2,119)= 23.58$, $p<.001$, $R^2=28.38\%$		
Predictor	b^a	*SE*	*t*
Constant	0.00	0.08	0.00
Moralization	-0.23	0.09	-2.62**
Negative attitude	-0.44	0.08	-5.33***
	DV = Equal treatment, $F(2,119)=36.31$, $p<.001$, $R^2=37.90\%$		
Predictor	b^a	*SE*	*t*
Constant	-0.00	0.08	-0.00
Moralization	-0.56	0.08	-7.46***
Negative attitude	-0.15	0.08	-1.82

Note. [a] Z-standardized regression coefficients; * $p < .05$, ** $p < .01$, *** $p < .001$.

Table 4 Supplement

Mediator model for simple mediation with reverse causation, all studies.

Study 1	DV = Social Cohesion (Mediator), $F(1,232)=4.13$, $p=.043$, $R^2=1.76\%$		
Predictor	b^a	*SE*	*t*
Constant	0.00	0.07	0.00
Moralization	-0.13	0.07	-2.03*
Study 2a	DV = Social Cohesion (Mediator), $F(1,764)=98.56$, $p<.001$, $R^2=11.43\%$		
Predictor	b^a	*SE*	*t*
Constant	0.00	0.03	0.00
Moralization	-0.34	0.03	-9.93***
Study 2b	DV = Social Cohesion (Mediator), $F(1,445)=50.58$, $p<.001$, $R^2=10.21\%$		
Predictor	b^a	*SE*	*t*
Constant	-0.01	0.05	-0.13
Moralization	-0.31	0.04	-7.11***
Study 3	DV = Social Cohesion (Mediator), $F(1,120)=10.89$, $p=.001$, $R^2=8.32\%$		
Predictor	b^a	*SE*	*t*
Constant	-0.08	0.08	-0.97
Moralization	-0.29	0.09	-3.30**

Note. Mediator here refers to the combined social cohesion indicator per study.

Note. [a] Standardized regression coefficients; $^{*}\ p < .05$, $^{**}\ p < .01$, $^{***}\ p < .001$.

Table 5 Supplement

Dependent variable models for simple mediation with reverse causation, all studies.

Study 1	DV = Stigmatization, $F(2,229)=16.42$, $p<.001$, $R^2=12.54\%$		
Predictor	b^a	*SE*	*t*
Constant	0.00	0.03	0.00
Moralization	0.17	0.04	2.72**
Social Cohesion	-0.29	.06	-4.64***
Study 2a	DV = Stigmatization, $F(2,763)=128.51$, $p<.001$, $R^2=25.20\%$		
Predictor	b^a	*SE*	*t*
Constant	0.00	0.03	0.00
Moralization	0.37	0.03	11.21***
Social Cohesion	-0.23	0.03	-6.99***
Study 2b	DV = Stigmatization, $F(2,444)=46.52$, $p<.001$, $R^2=17.32\%$		
Predictor	b^a	*SE*	*t*
Constant	0.01	0.04	0.13
Moralization	0.05	0.04	7.22***
Social Cohesion	-0.17	0.04	-3.76***
Study 3	DV = Stigmatization, $F(2,119)=15.88$, $p<.001$, $R^2=21.07\%$		
Predictor	b^a	*SE*	*t*
Constant	0.10	0.09	1.16
Moralization	0.19	0.09	2.10*
Social Cohesion	-0.40	0.09	-4.40***

Note. Dependent variables refer to the combined social cohesion measure per study.

Note. [a] Standardized regression coefficients; $^{*}\ p < .05$, $^{**}\ p < .01$, $^{***}\ p < .001$.
